# The Influence of the FFAR4 Agonist TUG-891 on Liver Steatosis in ApoE-Knockout Mice

**DOI:** 10.1007/s10557-023-07430-7

**Published:** 2023-01-27

**Authors:** Anna Kiepura, Maciej Suski, Kamila Stachyra, Katarzyna Kuś, Klaudia Czepiel, Anna Wiśniewska, Magdalena Ulatowska-Białas, Rafał Olszanecki

**Affiliations:** 1 Department of Pharmacology, Jagiellonian University Medical College, 16 Grzegorzecka Street, 31-531, Krakow, Poland; 2 Department of Pathomorphology, Jagiellonian University Medical College, 16 Grzegorzecka Street, 31-531, Krakow, Poland

**Keywords:** TUG-891, FFAR4/GPR120, Nonalcoholic fatty liver disease, Steatohepatitis

## Abstract

**Background:**

Nonalcoholic fatty liver disease (NAFLD) constitutes an independent risk factor for the development of coronary heart disease. Low-grade inflammation has been shown to play an important role in the development of atherosclerosis and NAFLD. Free fatty acid receptor 4 (FFAR4/GPR120), which is involved in damping inflammatory reactions, may represent a promising target for the treatment of inflammatory diseases. Our objective was to evaluate the effect of TUG-891, the synthetic agonist of FFAR4/GPR120, on fatty liver in vivo.

**Methods:**

The effect of TUG-891 on fatty liver was investigated in apoE^−/−^ mice fed a high-fat diet (HFD), using microscopic, biochemical, molecular, and proteomic methods.

**Results:**

Treatment with TUG-891 inhibited the progression of liver steatosis in apoE^−/−^ mice, as evidenced by histological analysis, and reduced the accumulation of TG in the liver. This action was associated with a decrease in plasma AST levels. TUG-891 decreased the expression of liver genes and proteins involved in de novo lipogenesis (Srebp-1c, Fasn and Scd1) and decreased the expression of genes related to oxidation and uptake (Acox1, Ehhadh, Cd36, Fabp1). Furthermore, TUG-891 modified the levels of selected factors related to glucose metabolism (decreased Glut2, Pdk4 and Pklr, and increased G6pdx).

**Conclusion:**

Pharmacological stimulation of FFAR4 may represent a promising lead in the search for drugs that inhibit NAFLD.

**Graphical abstract:**

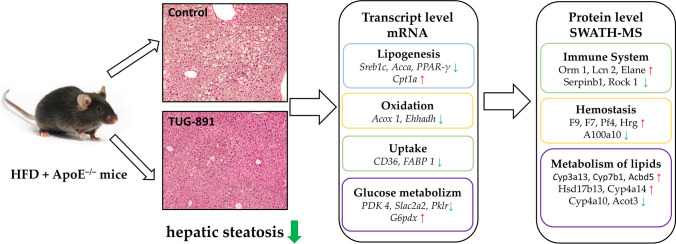

**Supplementary Information:**

The online version contains supplementary material available at 10.1007/s10557-023-07430-7.

## Introduction

Nonalcoholic fatty liver disease (NAFLD), characterized by excessive accumulation of triglycerides in hepatocytes, is the most common chronic liver disorder in the western world, and its prevalence is rapidly increasing globally [1, 2]. NAFLD can progress to inflammatory steatosis, known as nonalcoholic steatohepatitis (NASH), which can eventually lead to fibrosis, cirrhosis, and hepatocellular carcinoma [2, 3]. Steatosis of the liver is often accompanied by insulin resistance and obesity [4]. NAFLD is recognized as an early independent risk factor for the development of atherosclerosis; therefore, the identification of compounds that can inhibit liver steatosis may be an important step in the design of potential therapies for atherosclerosis and its complications [2, 5].

The excessive accumulation of triglycerides in the cytoplasm of hepatocytes was shown to be caused by an imbalance between cellular uptake/de novo synthesis of fatty acids and fatty acid oxidation (FAO), the latter marked by a reduction in the level of PPAR-α—the key transcription factor for liver FAO [6]. Glucose metabolism tightly regulated by insulin. Hepatic insulin resistance is marked by a failure of insulin to suppress hepatic glucose production, while at the same time stimulating increased lipogenesis. Elevated levels of circulating free fatty acids (FFAs), in part related to decreased insulin suppression of adipose tissue lipolysis, result in increased delivery of FFAs to the liver [7]. Therefore, the presence of liver steatosis is associated with a constellation of alterations in glucose, fatty acid, and lipoprotein metabolism, as well as mitochondrial dysfunction, which has been shown to aggravate the development of NAFLD [4].

In recent years, much attention has been focused on the action of free fatty acid receptors (FFARs), which have been shown to be expressed in hepatocytes and to impact metabolism [1, 8]. The type 4 receptor (FFAR4), with pleiotropic effects, is of particular interest [1, 9, 10]. FFAR4, also known as GPR120, acts as a receptor for long-chain polyunsaturated free fatty acids (PUFAs). It plays a key role in various physiological mechanisms including adipogenesis, appetite regulation, and regulation of food preferences [8]. Ichimura et al. showed that FFAR4-deficient mice fed a high-fat diet (HFD) developed obesity, glucose intolerance, and fatty liver, with increased hepatic lipogenesis [11, 12]. Oh et al. showed that activation of FFAR4 dampened inflammatory activation of macrophages [13]. The combination of the metabolic effects and the inhibition of inflammatory reactions have made FFAR4 stimulation an interesting area of investigation in the search for new drugs for the treatment/prevention of metabolic and circulatory disorders, but few data are available from animal models using FFAR4 agonists.

Recently, we demonstrated that activation of FFAR4 by its agonist, TUG-891, inhibited atherosclerosis in mice with knockout of apolipoprotein E (apoE^−/−^mice), which was associated with a beneficial phenotypical change between M1/M2 macrophages in atherosclerotic plaques [5]. ApoE^−/−^ mice are a well-known and widely used model for research on atherosclerosis and liver steatosis [14–16]. Unlike wild-type mice, they develop atherosclerosis on a chow diet, while liver steatosis still requires the use of a diet with higher fat content. However, the diet that causes homogeneous, moderate liver steatosis in apoE^−/−^ mice does not have to contain very high levels of fat (more than 20%) or cholesterol (ca. 1.25%), resulting in better model stability and homogeneity of results.

In this study we investigated the effects of TUG-891 on the development of hepatic steatosis in apoE^−/−^ mice fed an HFD, and identified possible pathways for this action using sequential window acquisition of all theoretical mass spectra (SWATH-MS) quantitative proteomics and molecular methods.

## Materials and Methods

### Animal Experiments

Female apoE-knockout mice of the C57BL/6J background strain were obtained from Charles River Laboratories (Calco, Lecco, Italy). Female apoE mice, widely used for pharmacological studies, were selected due to the homogeneity of this model: female apoE^−/−^ mice were shown to develop relatively greater and more homogeneous steatosis and atherosclerotic lesions than male apoE^−/−^ [17, 18]. All animal procedures were approved by the Jagiellonian University Ethical Committee on Animal Experiments (No. 167/2018). The animals were kept under a 12 h dark/12 h light cycle in air-conditioned rooms (22.5 ± 0.5 °C, 50 ± 5% humidity) with ad libitum access to water and food. The mice were put on an HFD (10% fat diet; 95 mg/kg cholesterol; Ssniff E15122-34, S8435-E014, ssniff Spezialdiäten GmbH, Soest, Germany) and treated with compound TUG-891 for 16 weeks beginning at the age of 8 weeks. The animals were randomly divided into two groups: the control group (apoE-knockout mice without treatment, on the same diet as above, *n* = 15) and the mice treated with TUG-891 (*n* = 15). TUG-891 (4-[(4-fluoro-4′-methyl[1,1′-biphenyl]-2-yl)methoxy]-benzenepropanoic acid; purity: 99.27%) (HY-100881, lot 23051, Prospecta, Poland) was administered subcutaneously (sc) at a dose of 20 mg/kg three times a week for 4 months. During the week, TUG-891 was administered to mice on Monday, Wednesday, and Friday, always between 8 and 9 am. Three separate vials (each containing a solution for all animals in the TUG-891 group for each day) were prepared weekly under sterile conditions and stored at −20 °C. The compound was dissolved in 50% dimethyl sulfoxide (DMSO) and 50% Kollisolv PEG [polyethylene glycol] E 300 50%. The manufacturer ensures the stability of the compound in the solvent at −20 °C for 1 month. Before injections, the solutions were heated to room temperature and vigorously vortexed for approximately 1 min. The control group received a vehicle DMSO 50% and a Kollisolv PEG E 300 50% prepared and stored in the same way. The daily dose of TUG-891 was chosen based on its positive effects at a dose of 35 mg/kg given intraperitoneally (ip) to study the influence of TUG-891 on body weight, in vivo energy metabolism, and FAO in the liver in mice; another study used a dose of 20 mg/kg once a day for 2 weeks, and the effects on food consumption, animal weight, and insulin resistance were studied and described in shorter murine experiments (20 days) [19–21]. Because in our setting TUG-891 was administered for a much longer period (16 weeks), the mixture of DMSO and Kollisolv was used to reduce the dose and potential toxicity of DMSO. At 6 months of age, the animals were injected with 1000 IU of Fraxiparine (nadroparin) ip (Sanofi, Paris, France) and euthanized by exposure to carbon dioxide. Blood was collected, and the aortas, hearts, and livers were dissected.

### Histology of the Liver

Liver tissue samples were fixed in formalin and embedded in paraffin, and 2 μm-thick paraffin sections were stained with hematoxylin-eosin (HE). All samples were evaluated by a pathologist with extensive experience in liver pathology. The analysis was performed using a single-blind method—the codes and assignment to groups were not known to the evaluating pathologist. Slides (three independent sections of each animal in at least 20 different fields of view) were microscopically evaluated for the presence and type of steatosis. The overall percentage of hepatocytes containing fat in the cytoplasm (as small droplets or a large drop) was estimated in the entire biopsy according to the SAF (steatosis-activity-fibrosis) scoring system for NAFLD: S0: < 3% of hepatocytes containing fat; S1: 3–33% of hepatocytes containing fat; S2: 34–66% of hepatocytes containing fat; S3: 66% of hepatocytes containing fat. In each field of view, the mean percentage of steatotic hepatocytes was specified. Preservation of the lobular structure, the presence of an inflammatory infiltrate in the lobular and portal areas, and the presence of necrotic hepatocytes were also assessed [22].

### Biochemical Methods

Plasma was centrifuged at 1000×*g* at 4 °C for 10 min and stored at −80 °C. Plasma levels of aspartate aminotransferase (AST) and alanine aminotransferase (ALT) were tested with the Reflovet Plus analyzer (Roche, Basel, Switzerland) using commercial kits (Reflotron GOT, Reflotron GPT, Roche, Basel, Switzerland). Triglyceride (TG) levels in the liver were evaluated using the Cayman Triglyceride Colorimetric Assay Kit (Cayman Chemical, Ann Arbor, MI, USA), according to the manufacturer’s instructions.

### Real-Time PCR

Total RNA was isolated from liver samples using the ReliaPrep™ RNA Tissue Miniprep System (Z6111, Promega, Madison, WI, USA), according to the manufacturer’s instructions. Quantitative real-time PCR using the GoTaq^®^ qPCR Master Mix (A600A, lot. 0000463011, Promega, Madison, WI, USA) was performed on the Bio-Rad CFX96 Touch™ Real-Time PCR system (Bio-Rad; Hercules, CA, USA). Commercially available primers (*Scd1*, *Srebp1*, *Fasn*, *Acca*, *Cpt1a*, *Acox1*, *Ehhadh*, *Cd36*, *Fabp1*, *Pdk4*, *G6pdx*, *Slc2a2*, *Pklr*, *Ppar-α*, *Ppar-γ* and *GAPDH*) from Bio-Rad (Hercules, CA, USA) were used to perform the real-time PCR reaction. Data analysis was performed using the 2^−ΔΔCt^ method with CFX Maestro software (Bio-Rad), and *GAPDH* expression was used as an internal control. The exact methodology of the RT-PCR reaction was described previously [23]. The numbers for analyses in particular methods were selected based on a priori statistical power analysis. In the case of PCR, the minimum number of *n* = 6 produced 80% power. The samples were randomly selected (based on a previously prepared randomized table).

### Liver Subcellular Fractionation

The liver was immediately subjected to fractionation to dissect the liver tissue into compartments to maximize the sensitivity and proteome coverage of the mass spectrometry measurements. The isolation of mitochondria-enriched and cytosolic fractions was carried out at 4 °C from freshly harvested mouse liver. Samples were collected and stored at −80 °C until assay. The exact methodology for liver fractionation was described previously [24].

### Sample Preparation for LC-MS/MS Analysis

The liver subcellular fractions were processed with the same sample preparation protocol. Isolated mitochondria were lysed in 500 μL of lysis buffer (2% SDS, 50 mM DTT in 0.1 M Tris-HCl pH 7.6), vortexed, incubated at 95 °C for 5 min, and clarified by centrifugation at 14,000×*g* for 10 min. The cytosolic fractions were concentrated and purified by overnight precipitation of acetone (1:6 sample-to-acetone ratio), then centrifuged at 14,000×*g* for 10 min. The protein pellets were then lysed in lysis buffer similar to mitochondrial specimens (buffer-to-tissue ratio of 10 [w/v]). Before protein digestion, the total protein concentration in the collected lysates was determined by tryptophan fluorescence assay [25]. Next, a volume containing 70 μg of total protein was transferred to Microcon-30kDa centrifugal filter units (Merck, Darmstadt, Germany), denaturated with 8 M urea and 0.1 M Tris-HCl pH 8.5, and digested into peptides using the filter-aided sample preparation (FASP) protocol [26]. Briefly, proteins were alkylated with iodoacetamide and cleaved with trypsin (Thermo Scientific, Waltham, MA, USA) with an enzyme-to-protein ratio of 1:50. Digestion of samples was carried out overnight in 50 mM Tris-HCl pH 8.5 at 37 °C. After digestion, peptide yields were determined using a WF test, and aliquots containing an equal amount of total peptides were desalted in C_18_ Ultra-Micro SpinColumns (Harvard Apparatus, Holliston, MA, USA). In one case (biological replicate of the cytosolic fraction in the control group), digestion failed to produce sufficient peptides to perform a successful SWATH measurement, and thus the latter was excluded from the analysis. The samples were then concentrated at a volume of ~ 5 μL and stored at −80 °C. For the preparation of project-specific spectral libraries (separately for mitochondrial and cytosolic fractions), equal amounts of peptides from all samples included in the analysis were combined and subjected to the fractionation protocol. HpH fractionation on C_18_ Micro SpinColumns (Harvard Apparatus, Holliston, MA) was carried out in 50 mM ammonium formate buffer (pH 10) with 12 consecutive injections of the eluent buffer, comprising 5, 10, 12.5, 15, 17.5, 20, 22.5, 25, 27.5, 30, 35 and 50% acetonitrile (ACN) in 50 mM ammonium formate buffer (pH 10), collected by centrifugation (300×*g*, 2 min), and dried in a SpeedVac concentrator (Eppendorf, Hamburg, Germany). In this way, the peptides were distributed across 12 HpH fractions separately for both subcellular fractions and analyzed by LC-MS/MS in data-dependent acquisition (DDA) mode for library generation. The latter was then used as a reference for sample measurements in SWATH acquisition mode. Before analysis, all samples and library peptide fractions were solubilized in 0.1% formic acid and 5% ACN at a concentration of 0.5 μg/μL and spiked with iRT [indexed Retention Time] peptide mix (Biognosys, Schlieren, Switzerland) to normalize retention time.

### Liquid Chromatography–Tandem Mass Spectrometry

Peptides (1 μg) were injected into a PepMap 100 RP C_18_ 75 μm i.d. × 25 cm column (Thermo Scientific, Waltham, MA, USA) through a trap column (PepMap 100 RP C_18_ 75 μm i.d. × 2 cm column; Thermo Scientific). For library generation, each peptide fraction was separated using a linear gradient of phase B 1% to 40% for 98 min (phase A—2% ACN and 0.1% FA; phase B—80% ACN and 0.1% FA) operating at a flow rate of 300 nL/min in an UltiMate 3000 HPLC system (Thermo Scientific) and applied to a TripleTOF 6600+ mass spectrometer (SCIEX, Framingham, MA, USA). The main working nanoelectrospray ion source (OptiFlow, SCIEX) was as follows: ion spray voltage 3 kV, interface heater temperature (IHT) 200 °C, ion source gas 1 (GS1) 10 and curtain gas (CUR) 25. For DDA, spectra were collected in full-scan mode (350–1400 Da), followed by 100 collision-induced dissociation (CID) MS/MS scans of the 100 most intense precursor ions of the previous full scan that exceeded an intensity of 100 counts per second under dynamic exclusion criteria. The samples analyzed in SWATH acquisition mode were separated using a phase 63 min of 1% to 40% B at a flow rate of 300 nL/min. For SWATH acquisition, spectra were collected in full-scan mode (400–1250 Da), followed by 100 SWATH MS/MS scans using a variable precursor isolation window approach, with m/z windows ranging from 6 to 90 Da and ion source settings similar to the DDA mode.

### Mass Spectrometric Raw Data Analysis, Spectral Library Generation, and SWATH Quantitation

The DDA data were searched against the mouse UniProt database (release 2020_1_1, 17,027 entries) using the Pulsar search engine implemented in Spectronaut software (Biognosys, Schlieren, Switzerland) [27] with default parameters (±40 ppm mass tolerance at the MS1 and MS2 levels, mutated decoy generation method, trypsin enzyme specificity). Deep learning-assisted iRT regression was set as the iRT reference strategy for RT to iRT calibration, with minimum *R*^2^ set to 0.8. The peptide, protein, and peptide–spectrum match (PSM) false discovery rate (FDR) were set to 1%. The library was generated using 3–6 fragment ions per precursor.

The project-specific library was then used to analyze the SWATH data in Spectronaut (Biognosys, Schlieren, Switzerland). Data were filtered with 1% FDR at the peptide and protein levels, while quantification and interference correction were performed at the MS2 level. Protein grouping was performed using the IDPicker algorithm [28]. Single-hit proteins were excluded from the analysis. The protein quantities were calculated by averaging the respective peptide intensities, while the latter were obtained as mean precursor quantities. The protein coefficients of variation (CVs) were calculated on the summed intensities of their respective peptides. Data were normalized using a global regression strategy, while statistical testing of differential protein abundance was performed using *t* tests with multiple-test correction after Storey [29]. The LC-MS data, library, results report, and Spectronaut project files have been deposited to the ProteomeXchange Consortium through the PRIDE partner repository [30] with the data set identifier PXD028535.

The functional grouping and pathway annotations were performed by PINE [31] using ClueGO [32] in the Cytoscape 3.7.2 software environment [33]. The CORUM-3.0 (release 03.09.2018), KEGG (release 17.02.2020), REACTOME (release 17.02.2020), and WikiPathways (release 17.02.2020) ontologies/routes were used in the analysis. The enrichment results were validated by an enrichment/depletion two-sided geometric statistical test with Bonferroni step-down as the *p*-value correction method. The minimum and maximum gene ontology (GO) levels were established as 1 and 4, respectively, with cluster criteria of a minimum of five genes comprising a minimum of 2% of the GO terms. The kappa score threshold was set at 0.4.

### Statistical Analysis

The results shown are mean ± standard error of the mean (SEM) of multiple experiments. The equality of variance and normality of the data were checked using the Kolmogorov–Smirnov and Shapiro-Wilk tests; then the nonparametric Mann–Whitney *U* test or *t* test, when appropriate, was used for statistical analysis of the data. A value of *p* < 0.05 was considered statistically significant.

## Results

### The Influence of TUG-891 on Hepatic Steatosis

Hematoxylin-eosin staining showed changes in liver structure in apoE^−/−^ mice fed an HFD. The cytoplasm of hepatocytes in the liver of apoE^−/−^ mice on HFD (control) had a granular structure with signs of macrovesicular steatosis in approximately 30% of the hepatocytes. Microvesicular steatosis was present in less than 5% of the hepatocytes.

**Fig. 1:**
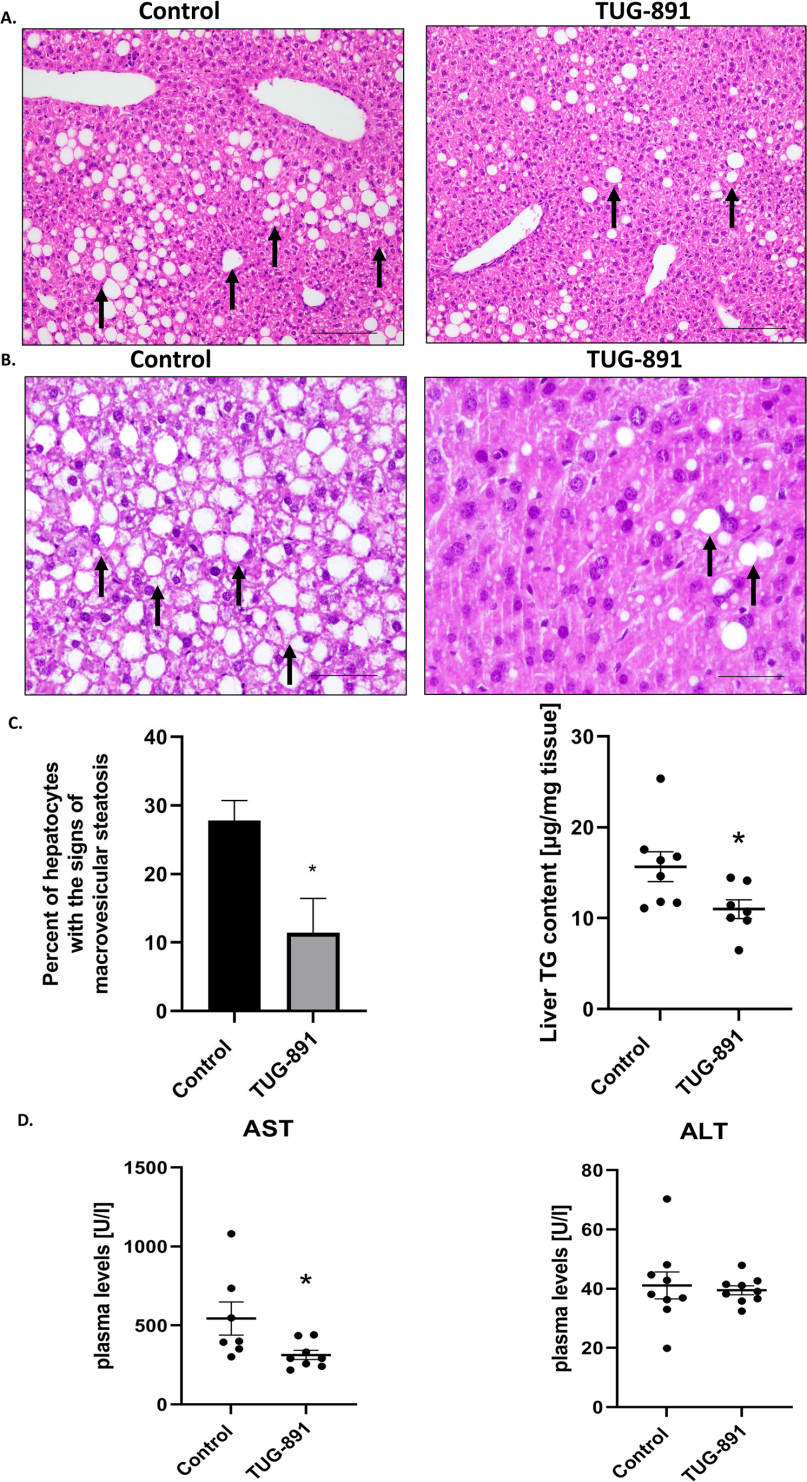
Influence of TUG-891 on hepatic steatosis in apoE^−/−^ mice. Representative images of the livers of apoE^−/−^ mice on an HFD and TUG-891-treated apoE^−/−^ mice on an HFD; the images show hematoxylin and eosin staining and quantitative analysis of macrovesicular steatosis (**A**, **C**; arrows: steatosis cells, magnification: ×20, scale bars = 50 μm), inset: H&E staining at ×40 provides greater insight into the nature of steatosis in areas of its high intensity in tissue; only macrovesicular steatosis is visible (**B**) as well as TG content (**C**) in the liver and plasma levels of alanine aminotransferase (ALT) and aspartate aminotransferase (AST) in apoE^−/−^ mice (**D**) (mean ± SEM; * *p* < 0.05 as compared to apoE^−/−^ mice on the HFD)

The portal spaces (containing the portal duct or ducts, the portal vein, and the hepatic artery) were not enlarged and did not present inflammatory infiltrates or necrotic changes. Macrovesicular steatosis was evident in approximately 10% of hepatocytes in the TUG-891-treated animals (Fig. 1A). TUG-891 did not influence the percentage of cells with microvevsicular changes. Administration of TUG-891 resulted in a significant decrease in the level of triglycerides in the liver of apoE^−/−^ mice (Fig. 1B). TUG-891 significantly decreased plasma levels of AST and did not significantly influence ALT (*p* = 0.7) in apoE^−/−^ mice on HFD (Fig. 1C). As we reported in the previously published work on the development of atherosclerosis of these exact same animals, there were no differences between the control and TUG-891-treated animals in plasma lipids [5].

### Effects of TUG-891 on mRNA Expression in the Liver

**Fig. 2:**
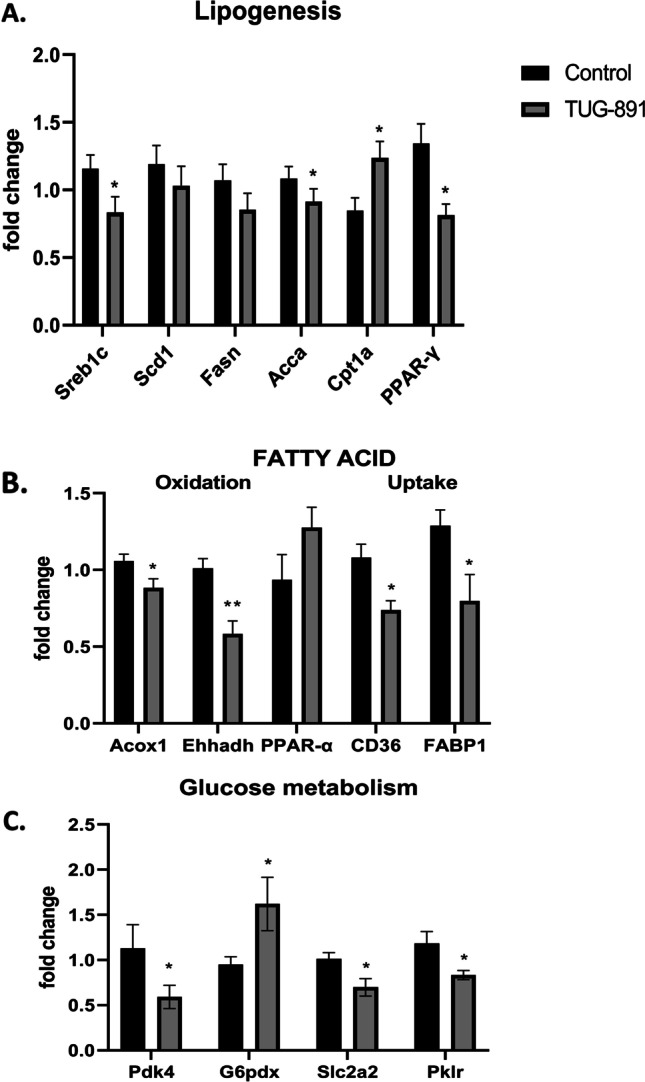
The mRNA expression of selected factors related to lipogenesis [*n* = 6–7 per group] (**A**), fatty acid oxidation and uptake [*n* = 6–7 per group] (**B**), and glucose metabolism [*n* = 5–7 per group] (**C**) in the liver of control and TUG-891-treated mice. Data are mean ± SEM (* *p* < 0.05, ** *p* < 0.01 as compared to control mice)

**Fig. 3:**
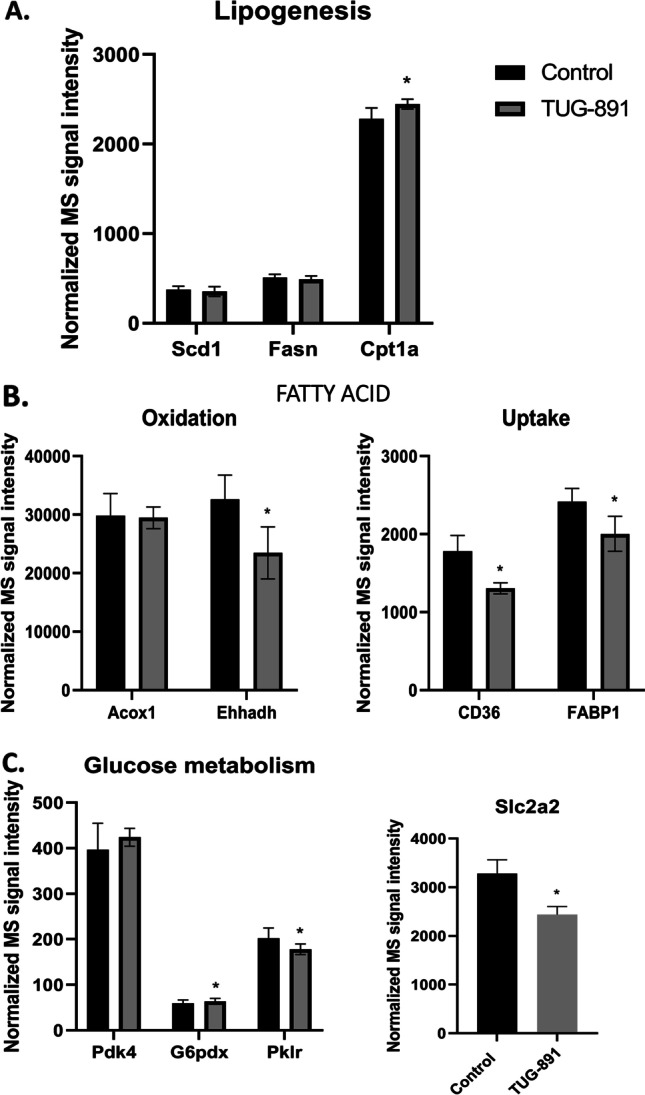
Proteomic analysis of selected factors related to lipogenesis (**A**), fatty acid oxidation and uptake (**B**), and glucose metabolism (**C**) in the liver of control and TUG-891-treated mice. The quantity data extracted from the SWATH data set (* *p* < 0.05, ** *p* < 0.01; *n* = 7–8 per group)

We evaluated the influence of TUG-891 on the expression of selected factors associated with lipogenesis, uptake and oxidation, and glucose metabolism in the liver. The levels of *Srebp-1c* and *Acca* mRNA decreased significantly in the animals compared to those of the control group, in contrast to the level of Cpt1a mRNA which increased significantly (Fig. 2A); the relative level of *Fasn*, *Scd*, and *Ppar-γ* tended to be lower, but the difference did not reach statistical significance (*p* = 0.1847; *p* = 0.4; *p* = 0.0786). The expression of oxidation-related factor mRNA (*Acos1*, *Ehhadh*) was significantly decreased in the TUG-891-treated group (Fig. 2B); expression of *Ppar-α* mRNA was relatively increased, but the difference did not reach statistical significance (*p* = 0.1677; Fig. 2B). The mRNA levels of the molecules associated with fatty acid uptake—*Cd36* and *FABP1*—were significantly reduced in the TUG-891 group (Fig. 2B). The expression of genes related to glucose metabolism, including glucose transporter 2 (*Glut2*, codified by the *Slc2a2* gene), pyruvate dehydrogenase kinase 4 (*Pdk4*), and pyruvate kinase L/R (*Pklr*), was significantly reduced and the glucose-6-phosphate 1-dehydrogenase X (*G6pdx*) gene was significantly increased after treatment with TUG-891 (Fig. 2C). The mRNA data were validated by SWATH-MS proteomic assessment (Fig. 3A–C). In contrast to PCR data, mass spectrometry-based measurements not only provide an estimate of differences in protein abundance in the experimental groups but also indicate the quantitative relation of the particular proteins of interest. This information can be used as an indication of key molecular nodes of the biological processes modulated by drug administration.

### Proteomic Profiling from Liver of ApoE ^−/−^ Mice

DDA mass spectrometry measurements resulted in the identification of 49,543 and 32,338 proteotypic peptides, which allowed the preparation of a spectral library comprising 5620 and 3788 protein groups (including 799 and 627 single-hit proteins) for mitochondrial and cytosolic fractions, respectively. The libraries were then used to analyze the SWATH data sets with Spectronaut. Recovery of the spectral library was 62.3% and 69% with data between protein group profiles of 88.2% and 92.4% for the mitochondrial and cytosolic fractions, respectively (Supplemental Fig. 1). The median CVs of the protein groups were calculated in the range of 16.8–30.5% for all experimental groups in both fractions (Supplemental Fig. 1), allowing the estimation of a significant quantitative cutoff for the absolute 1.5- and 1.4-fold change for the mitochondrial and cytosolic fractions, respectively (statistical power 97.5% and 98.5% for the mitochondrial and cytosolic fractions, respectively). Estimates of the reproducibility of LC-MS analyses and quantitation quality are collected in Supplementary Fig. 2.

**Fig. 4:**
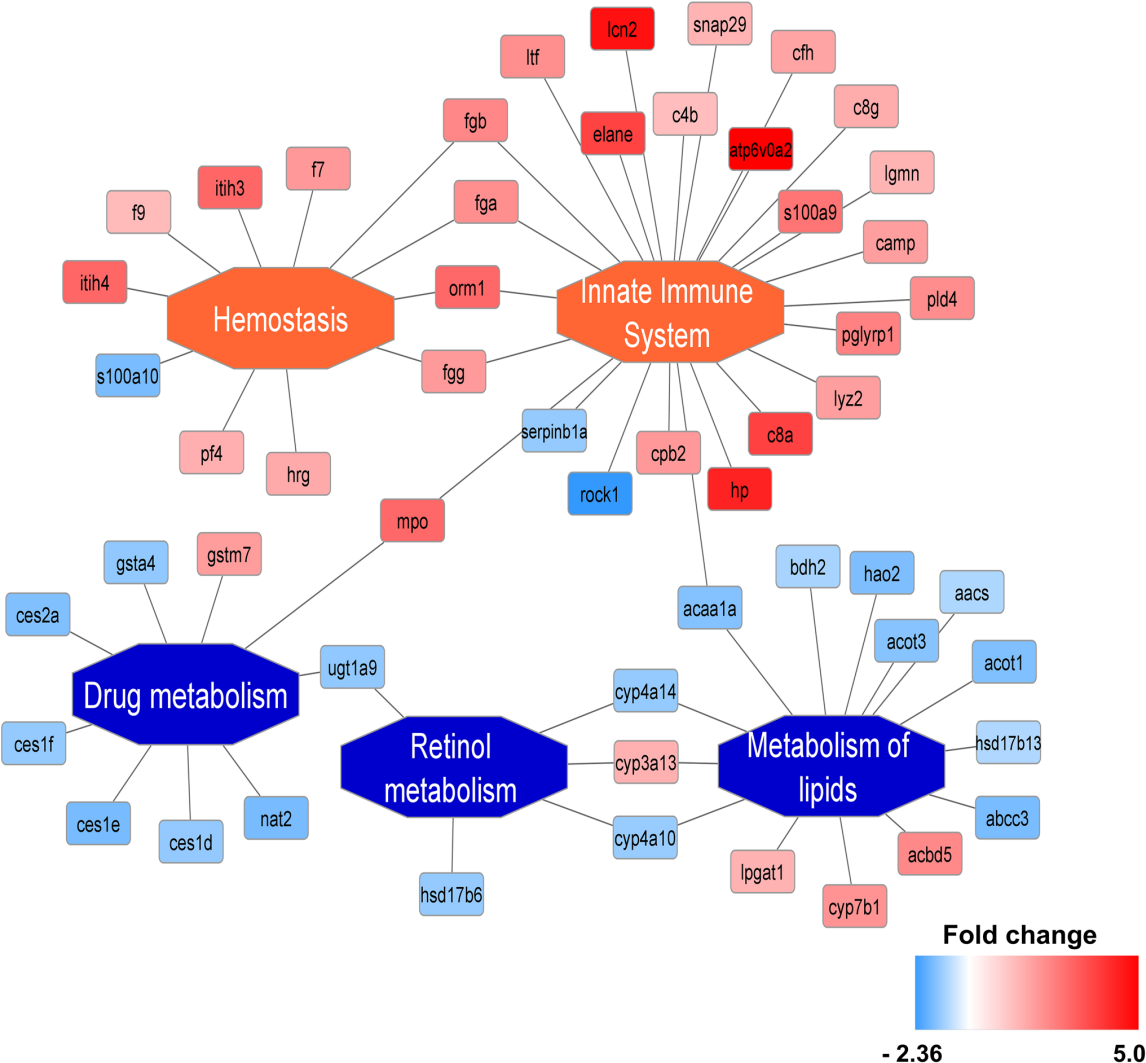
Pathway enrichment of differentially regulated proteins in TUG-891-treated apoE^−/−^ mice. Changes in the abundance of particular proteins enriched in the biological process are used as an approximation of the latter effective difference evoked by TUG-891 administration. Proteins in red were induced while those in blue were repressed in the FFAR4 agonist group as compared to controls. The legend provides the details regarding the magnitude (fold change) of the observed changes

On average, the acquired data enabled the identification and quantification of 3095 and 2420 protein groups, of which 92 and 47 were significantly regulated in liver mitochondria and cytosol, respectively (Supplemental Table 1). These proteins were then collectively included in the pathway analysis. Their differential abundance was used as a proxy for the TUG-891-induced changes in the efficacy of the biological process in which they participate in the livers of apoE^−/−^ mice (Fig. 4).

## Discussion

In this study, we demonstrated that TUG-891 significantly inhibited the development of liver steatosis in apoE^−/−^ mice fed a western diet. To our knowledge, this is the first study to report that pharmacological stimulation of FFAR4 may effectively inhibit hepatic steatosis in vivo. We found that TUG-891 treatment resulted in a 20% reduction in the number of hepatocytes with signs of steatosis and a significant reduction in liver TG levels. Such effects were accompanied by hepatoprotective action, as evidenced by the decrease in plasma AST levels. These results represent an important continuation of our previous study showing that TUG-891 significantly inhibited the development of atherosclerosis in the very same experimental setting, in which it did not induce significant changes in the blood lipid levels or weight of the animals, and no toxic effects of the compound were observed [5].

Our results suggest that the inhibitory effect of TUG-891 on the development of fatty liver is associated with inhibition of the de novo lipogenesis (DNL) pathway, as evidenced by a significant reduction in the expression of *Srebp-1c* mRNA and acetyl-CoA carboxylase (*Acc*) mRNA/protein. Several studies point to Srebp-1 as a key regulator of DNL [34]. The liver triglyceride level was shown to be higher in transgenic mice [35]. Furthermore, SREBP-1 is elevated in the liver of patients with NAFLD. There is also a correlation between SREBP-1c expression and the severity of insulin resistance and obesity in patients with metabolic syndrome [2]. ACC is the first enzyme involved in the DNL pathway, and its cytosolic isoform ACC1 is involved in the rate-limiting step in DNL (ACC2, a mitochondrial membrane-associated enzyme, is involved in FAO). DNL has been shown to be inhibited in the liver of ACC1-knockout mice [36]. In this context, further studies of the molecular mechanisms responsible for the TUG-891-dependent decrease in liver expression of *Srebp-1c* and *Acc1* appear to be a very interesting prospect.

In our model, the influence of TUG-891 on other important factors related to DNL consistently indicated its inhibitory effect on this metabolic pathway in the liver: increased *Cpt1a* and decreased *Ppar-γ* levels. The carnitine palmitoyltransferase (CPT) system is responsible for the transport of long-chain fatty acids from the cytoplasm to mitochondria, where the fatty acids undergo β-oxidation. Decreased CPT1A expression was shown to be involved in the development of NAFLD in a mouse model with NAFLD [37]. The nuclear receptor PPAR-γ, mainly due to its role in the processes of differentiation and modulation of inflammatory responses of various cells, also has a modulatory effect on DNL in the liver [38]. PPAR-γ deletion in mouse hepatocytes has been shown to protect against the development of steatosis in mice with liver steatosis and diabetes. Therefore, the increase in CPT1A and the decrease in PPAR-γ induced by TUG-891 seem to suggest its broad inhibitory effect on liver DNL [36].

In our setting, TUG-891 treatment resulted in a decrease in the expression of genes related to fatty acid uptake and oxidation—*Cd36* and *Fabp1*, and *Ehhadh* and *Acox1*, respectively [39]. As shown, CD36-knockout mice were protected against HFD-induced liver steatosis and reduced expression of FABP1 resulting in decreased intracellular lipid-binding capacity and reduced lipotoxicity. The influence of TUG-891 on factors associated with these pathways may also play a mechanistic role in reducing liver steatosis, but this requires further research.

The development of fatty liver has been shown to be associated with abnormal glucose metabolism in hepatocytes [40]. In our model, TUG-891 treatment resulted in changes in liver expression of factors related to glucose metabolism in the liver. Glucose transporter 2 (GLUT2, coded for by the *Slc2a2* gene), *Pdk4*, and *Pklr* were decreased and *G6pdx* mRNA increased after treatment with TUG-891. The interpretation of the influence of TUG on the development of NAFLD in the context of its influence on factors related to glucose metabolism is ambiguous. Our results on the expression of GLUT-2 seem to contradict the observations so far: Plasma levels of FFAs, as well as their uptake into hepatocytes, were reported to increase in GLUT2-deficient mice [41]. On the other hand, our results for PKLR and PDK4 appear to indicate a beneficial effect of TUG-891 on hepatic glucose metabolism in the context of the development of steatosis, as PKLR and PDK4 were shown to aggravate NAFLD [42, 43]. Therefore, the mechanistic links between the influence of TUG-891 on factors related to glucose metabolism and its potential importance for the antisteatotic action of TUG-891 in the liver are unclear.

In general, many mechanisms (that is, related to inflammatory stimulation, mitochondrial dysfunction, reactive oxygen species [ROS] production, and endoplasmic reticulum [ER] stress) at the cellular and intracellular levels are believed to play a role in the development of NAFLD [44]. Consequently, the action of FFAR4 appears to be pleotropic and largely cell-dependent [8].

Mitochondria are considered key organelles and are at the crossroads of many pathways that play a role in the development of the fatty liver. Mitochondria are involved not only in cellular respiration, but also in gluconeogenesis and other key biosynthetic activities, as well as contributing to the oxidative stress and inflammatory reactions observed in NAFLD. Our objective was to thoroughly detect possible changes in mitochondrial/cytosolic pathways in hepatocytes after treatment with TUG-891 using a quantitative proteomics method, SWATH-MS proteomics. To maximize the sensitivity and proteome coverage of the measurements, we employed a subcellular fractionation of liver tissue prior to SWATH-MS analysis, as sample fractionation at the peptide level is not recommended in data-independent acquisition (DIA) measurements. One of the main advantages of SWATH (DIA) data sets is high reproducibility with low CV values. The sample fractionation process at the peptide level introduces additional variability into the sample preparation protocol, which decreases the quality of the SWATH data set. In principle, increased coverage in a SWATH analysis is achieved by optimizing the chromatographic separation and mass spectrometry acquisition methods and building a better spectral library. To ensure the highest quantitative data output, we prepared separate spectral libraries for both fractions instead of one for the entire tissue lysate. In this way, we were able to assess the quantitative changes in protein induced by TUG-891 administration in approximately 6500 unique protein groups. Furthermore, we were able to focus our quantitative protein analysis on the cytosolic and mitochondrial compartments. The latter was especially important given that mitochondrial dysfunction is a well-described characteristic of NAFLD. As a result, we revealed some interesting clues for further research on the mechanisms of action of TUG-891. Furthermore, we validated several observations from mRNA measurements at the protein level. Quantitative data were of high quality (Supplementary Fig. 2) and all proteins of interest were specifically enriched in one of the fractions. Therefore, our SWATH data set represents robust and comprehensive proteomic insight into TUG-891-induced changes in the liver of apoE^−/−^ mice. In addition, taking into account the longitudinal nature of the experimental design of the present study and the relative homogeneity of liver tissue, the quantitative changes observed in transcripts and proteins constitute the molecular fingerprint of the prolonged action of the FFAR4/GPR120 agonist.

Analysis of the liver proteome revealed the effect of TUG-891 on the expression of many proteins related to lipid/xenobiotic metabolism, homoeostasis, and immunity.

Myo-inositol was reported to lower serum lipid levels and reduce the risk of fatty liver, and inositol synthesis may contribute to NAFLD [45]; therefore, the increase in inositol-3-phosphate synthase 1 (ISYNA1), involved in inositol synthesis, could potentially play a role in the TUG-891-dependent inhibition of liver steatosis.

Increasing evidence suggests that some members of the CYP450 family, mainly due to the aggravation of oxidative stress, may contribute to the pathogenesis of NAFLD and NASH [46]. Interestingly, TUG-891 downregulated the 17-β hydroxysteroid dehydrogenase 13 (HSD17B13), an important regulator of the biogenesis, growth, and degradation of lipid droplets in the liver [46]. Notably, several loss-of-function mutations in the human HSD17B13 gene have been shown to confer strong anti-inflammatory and antifibrotic effects in the liver, and a few single-nucleotide polymorphisms in the HSD17B13 gene have also been associated with the development of NAFLD in humans [47]. This interesting clue on the potential mechanism of antisteatotic action of TUG-891 in the liver needs to be verified.

Somewhat surprisingly, massive changes in proteins that regulate oxidative stress did not change with TUG-891. The TUG-891-dependent upregulation of STEAP4 may be interesting in this regard, since metalloprotease STEAP4, a member of the family of six transmembrane proteins, has been recognized as a modulator of inflammation and nutrient metabolism [48]. The loss of STEAP4 in mice leads to increased inflammatory cytokine production in visceral white adipose tissue and systemic insulin resistance. Furthermore, STEAP4 overexpression significantly increased SOD2 expression and mitochondrial antioxidant capacity, ultimately reducing cellular oxidative stress. In the context of the protective effect on mitochondria, the mechanism linking FFAR4 and STEAP4 may be interesting and worthy of further research. Additional research is also warranted on results showing that TUG-891 increases the production of the main urinary proteins (MUP-6, MUP-11, MUP-18) in the liver, which, as studies increasingly indicate, may have interesting functions in the regulation of metabolism [49].

Some of our proteomics results indicated increased liver production of proteins involved in coagulation (coagulation factor VII and IX, platelet factor 4, heavy chain 3 and 4, and fibrinogen alpha/beta/gamma chain) and inflammatory processes of innate immunity (e.g., orosomucoid 1, selected complement components, neutrophil gelatinase-associated lipocalin, myeloperoxidase) in the liver after treatment with TUG-891. Such results may come as a surprise in the context of reports of the mutual involvement of inflammatory reactions and thrombosis (often referred to by the common term thromboinflammation) in many cardiovascular pathologies [50]. In particular, in our setting, we did not observe any thrombotic complications or increased plasma levels of biochemical markers of inflammation in blood serum [5]. Certainly, these aspects of the action of TUG-891 in vivo require further research.

Our work indicates for the first time the steatosis-inhibiting effect of the FFAR4/GPR120 agonist, but we cannot ignore the limitations of this study. The difficulties in interpreting proteomics results and the need for their further verification in studies of cause-and-effect relationships constitute the most obvious limitation of our work. An important addition to our research would be the metabolomic data that confirm the effect of TUG-891 on the lipogenesis and lipolysis pathways in the liver. Despite the well-documented selectivity of TUG-891 for FFAR4/GPR120, its possible off-target effects in vivo could be verified by the concomitant administration of the FFAR4/GPR120 antagonist (e.g., AH-7614). The experiment should also be repeated on other NAFLD models and supported by histological methods that will show even more accurately the effects of TUG-891 on steatosis and possible fibrosis (e.g., Oil Red O and Masson’s trichrome staining). Furthermore, it would be very interesting to compare the potency of TUG-891 with known substances that inhibit steatosis (for example, metformin) in the same model. Thus, it is clear that great caution must be exercised when translating the results from animal models to human pathology.

## Conclusions

In this study we showed for the first time in an in vivo model that the use of the synthetic FFAR4 stimulator (TUG-891) resulted in a significant reduction in fatty liver. This activity of TUG-891 was mainly related to its inhibitory effect on the expression of important factors that play a role in the liver DNL pathway, as well as in the uptake and oxidation of fatty acids. Quantitative proteomics indicated that several other possible mechanisms of TUG-891 will be verified in the next studies. FFAR4 stimulation of FFAR4 by synthetic agonists appears to represent a promising lead in the search for drugs that inhibit NAFLD.

### Supplementary Information

Supplemental Fig. 1 Summary of the identification of protein groups. Protein group identification details in all of the LC-MS runs (A). Spectral library recovery (B) and data completeness (C). Coefficient of variations for protein groups under experimental conditions (D). (PPTX 581 kb)

Supplemental Fig. 2 Summary of protein group quantification. The TIC overlay of all LC-MS runs and iRT elution profiles showed excellent separation reproducibility (A), while normalization of the data allowed for reliable and accurate quantitation, as evidenced by the symmetrical histogram (B). (PPTX 394 kb)

ESM 1 (XLSX 32 kb)

## Data Availability

The datasets generated and/or analysed during the current study are available from the corresponding author on reasonable request.

## Abbreviations

*ACCA*: Acetyl-CoA carboxylase
*ALT*: Alanine aminotransferase
*AST*: Aspartate aminotransferase
*CD36*: *CD36* molecule
*cDNA*: Complementary DNA
*DNL*: De novo lipogenesis
*FABP1*: Fatty acid-binding protein 1
*FAO*: Fatty acid oxidation
*FASN*: Fatty acid synthase
*FFA*: Free fatty acids
*FFAR4/GPR120*: Free fatty acid receptor 4
*GAPDH*: Glyceraldehyde 3-phosphate dehydrogenase
*HE*: Hematoxylin/eosin
*HFD*: High-fat diet
*mRNA*: Messenger ribonucleic acid
*NAFLD*: Nonalcoholic fatty liver disease
*NASH*: Nonalcoholic steatohepatitis
*PPAR-α*: Peroxisome proliferator-activated receptor alpha
*PPAR-γ*: Peroxisome proliferator-activated receptors gamma
*PUFA*: Long-chain polyunsaturated fatty acids
*RT-PCR*: Reverse transcription polymerase chain reaction
*SCD1*: Stearoyl-CoA desaturase-1
*SREBP-1c*: Sterol regulatory element-binding protein 1c
*TG*: Triglyceride
*TUG-891*: 4-[(4-Fluoro-4′-methyl[1,1′-biphenyl]-2-yl)methoxy]-benzenepropanoic acid
