# Supplementary figures and images for: The Influence of the FFAR4 Agonist TUG-891 on Liver Steatosis in ApoE-Knockout Mice
Source: Cardiovasc Drugs Ther. 2023 Jan 27;38(4):667–78. doi: 10.1007/s10557-023-07430-7 (PMC11266261; doi:10.1007/s10557-023-07430-7)

## Slide 1
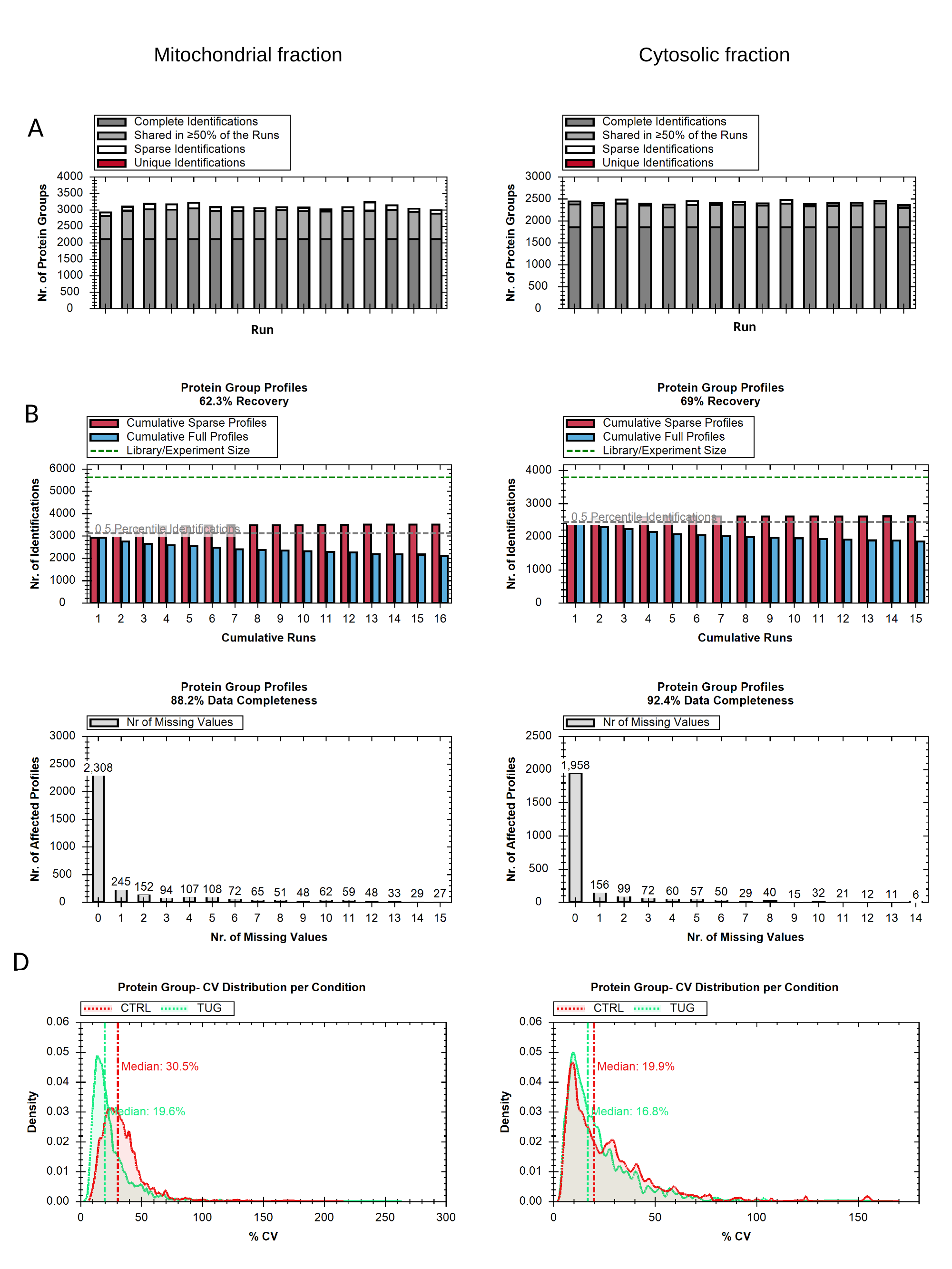

Mitochondrial fraction
Cytosolic fraction
A
Run
Run
B
C
D

Supplement: Supplementary file 1 — Summary of the identification of protein groups. Protein group identification details in all of the LC-MS runs (A). Spectral library recovery (B) and data completeness (C). Coefficient of variations for protein groups under experimental conditions (D). (PPTX 581 kb) [file 10557_2023_7430_MOESM1_ESM.pptx]

## Slide 1
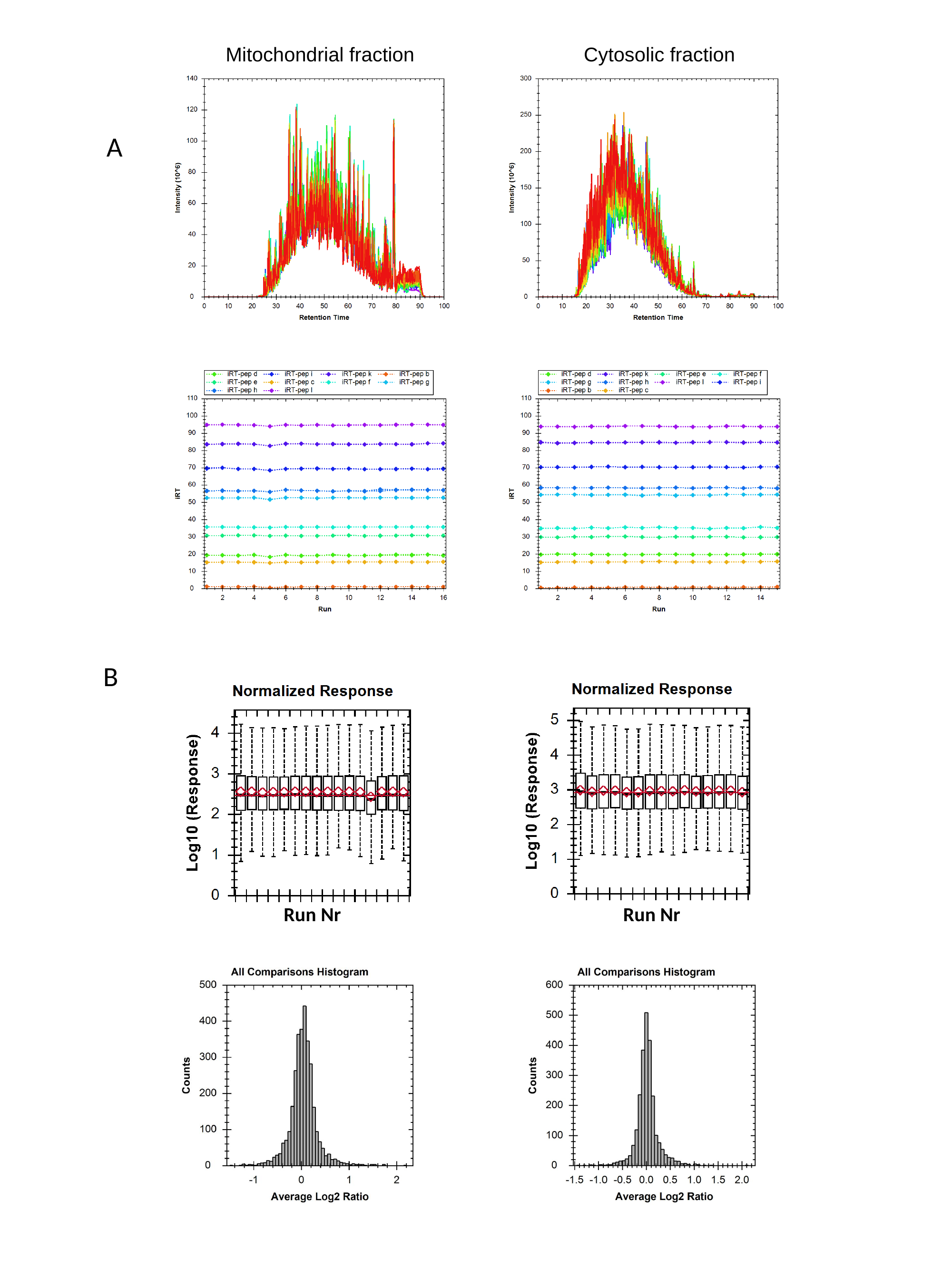

Mitochondrial fraction
Cytosolic fraction
A
B
Run Nr
Run Nr

Supplement: Supplementary file 2 — Summary of protein group quantification. The TIC overlay of all LC-MS runs and iRT elution profiles showed excellent separation reproducibility (A), while normalization of the data allowed for reliable and accurate quantitation, as evidenced by the symmetrical histogram (B). (PPTX 394 kb) [file 10557_2023_7430_MOESM2_ESM.pptx]
